# Improved accuracy in flow mapping of congenital heart disease using stationary phantom technique

**DOI:** 10.1186/1532-429X-11-S1-P102

**Published:** 2009-01-28

**Authors:** Thomas A Miller, Adrian M Moran

**Affiliations:** Maine Medical Center, Portland, ME USA

**Keywords:** Congenital Heart Disease, Cardiovascular Magnetic Resonance, Bicuspid Aortic Valve, Flow Mapping, Branch Pulmonary Artery

## Objective

We aimed to define the differences between flow mapping with and without baseline correction using a stationary phantom when evaluating congenital heart disease (CHD).

## Background

Flow mapping by cardiovascular magnetic resonance (CMR) has become the gold standard for defining cardiac output, shunt flow and regurgitation. Previous reports have highlighted the presence of inherent errors in flow mapping by CMR secondary to eddy currents and concomitant gradients. The use of a stationary phantom to control for these errors can improve predicted Qp/Qs ratios in normal volunteers. To our knowledge, no one has investigated the effect of phantom correction in CHD.

## Methods

All patients were imaged using GE 1.5 T Signa HDX systems. Phase velocity flow mapping by CMR was performed when clinically indicated on 20 patients with repaired Tetrology of Fallot (ToF), 7 patients with atrial level shunting (ASD/PAPVR), and 13 patients with bicuspid aortic valves (BAV). Flow velocities through the ascending aorta, main pulmonary artery (MPA) and proximal branch pulmonary arteries (BPA) were calculated using commercially available software (ReportCard) by placing a region of interest (ROI) around the vessel lumen. Flow velocities were then corrected by measuring baseline velocities in a ROI of the same size and location on a corresponding stationary phantom image. Comparison was then made between the uncorrected and corrected flows, degree of shunting, and regurgitant fraction. Qp/Qs was calculated as the ratio of net BPA flow to aortic flow.

## Results

Marked variation in net flow was seen in all vessels (Table 1). The variation in net flow was largest when analyzing the MPA/RVOT with a range of 0.2 to 70 ml/min (0.3–107.5% difference). In repaired ToF patients, the average Qp:Qs ratio was 1.03 with phantom correction and 1.15 without. Based on previously published criteria, 26% of the ToF group would have been incorrectly classified as having a residual shunt without performing phantom correction. In the ASD/PAPVR group 100% had significant shunting with phantom correction, while only 71% of uncorrected flows detected significant shunting. In the BAV group, the differences in the regurgitant fraction ranged from 0 to 31%. 23% of the patients changed their classification between mild, moderate and severe regurgitation when the phantom was performed.

**Table 1:** Differences in net flows between corrected and uncorrected flow mapping

	Aorta	MPA/RVOT	RPA	LPA
Avg difference in net flow (ml/min)	12.1	16.8	3.4	6.6
Range of difference in net flow (ml/min)	0.0–68.4	0.2–70	0.1–8.3	0.1–23.1
%Difference in net flow (diff/corrected net flow)	13%	25%	8%	20%
Range of %difference	0–62%	0.3–108%	0.3–24%	0.3–59%

## Conclusion

The impact of inherent errors in CMR flow mapping should not be underestimated. The range of variation, in common CHD, appears large and varied. Failure to correct for such variation can lead to clinically significant misinterpretation of flow data. The use of the stationary phantom correction technique appears to improve flow accuracy in patients with CHD. Further research is needed to elicit the causes and best control of such errors.

